# The lncRNA BDNF-AS is an epigenetic regulator in the human amygdala in early onset alcohol use disorders

**DOI:** 10.1038/s41398-019-0367-z

**Published:** 2019-02-06

**Authors:** John Peyton Bohnsack, Tara Teppen, Evan J. Kyzar, Svetlana Dzitoyeva, Subhash C. Pandey

**Affiliations:** 1 Center for Alcohol Research in Epigenetics, Department of Psychiatry, University of Illinois at Chicago, Chicago, IL 60612 USA; 2 Jesse Brown VA Medical Center, Chicago, IL 60612 USA; 3 Department of Anatomy and Cell Biology, University of Illinois at Chicago, Chicago, IL 60612 USA

## Abstract

Adolescent alcohol drinking is known to contribute to the development and severity of alcohol use disorders (AUDs) later in adulthood. Recent studies have shown that long non-coding RNAs (lncRNAs) are critical for brain development and synaptic plasticity. One such lncRNA is natural occurring brain-derived neurotrophic factor antisense (*BDNF-AS*) that has been shown to regulate BDNF expression. The role of *BDNF-AS* lncRNA in the molecular mechanisms of AUD is unknown. Here, we evaluated the expression and functional role of *BDNF-AS* in postmortem amygdala of either early onset or late onset alcoholics (individuals who began drinking before or after 21 years of age, respectively) and age-matched control subjects. *BDNF-AS* expression is increased in early onset but not in late onset AUD amygdala and appears to be regulated epitranscriptomically via decreased *N*6-methyladenosine on *BDNF-AS*. Upregulation of *BDNF-AS* is associated with a significant decrease in BDNF expression and increased recruitment of EZH2, which deposits repressive H3K27 trimethylation (H3K27me3) at regulatory regions in the *BDNF* gene in the early onset AUD group. Drinking during adolescence also contributed to significant decreases in activity-regulated cytoskeleton-associated protein (ARC) expression which also appeared to be mediated by increased EZH2 deposition of repressive H3K27me3 at the *ARC* synaptic activity response element. These results suggest an important role for *BDNF-AS* in the regulation of synaptic plasticity via epigenetic reprogramming in the amygdala of AUD subjects who began drinking during adolescence.

## Introduction

Alcohol use disorders (AUDs) are chronic, debilitating psychiatric illnesses that contribute to considerable socioeconomic and health burden worldwide^1^. Age of onset is one of the primary risk factors for the development of AUDs, as individuals who began drinking during adolescence are four times more likely to develop AUDs in adulthood^2^. Adolescence is a critical period in brain development and adolescent drinking decreases orbitofrontal cortex activity and increases amygdala activity^3^ leading to less executive control, more emotional impulsivity, alterations in decision-making, and have a higher risk to engage in risky behaviors and develop mental health problems later in life^4,5^. The amygdala acts as a hub for the processing of sensory signaling from cortical regions, and is involved in regulation of emotional reactivity, decision-making, and negative affective state of alcohol addiction^6,7^. During adolescence, the amygdala undergoes several key changes in specific inter- and intra-region connectivity^8^, and this process is thought to be disrupted by alcohol use^9^. Recent studies have begun to examine the molecular basis of these changes in the amygdala in preclinical models of adolescent alcohol exposure and have identified numerous potential molecular components, such as brain-derived neurotrophic factor (BDNF), that are involved in the development of alcohol dependence in adulthood^10–12^.

The neurotrophin BDNF has emerged as a critical molecular player in central nervous system (CNS) development, psychiatric disorders, and AUDs^13–15^. BDNF is associated with survival and differentiation of neurons, activity-dependent neuroplasticity, long-term potentiation, learning, memory, and anxiety^13,16^. BDNF is critical for normal brain development, as constitutive knockout mice die during second week after birth^13^ and is known to be involved in synaptogenesis and maintenance of dendrites during development and adulthood^13,17^. BDNF regulates synaptic plasticity through interactions with the tropomyosin-related kinase B (TrkB) receptor and induction of several signaling cascades that are involved in the regulation of synaptic plasticity and cAMP-responsive element-binding protein (CREB)-dependent gene expression^13^. Decreased expression of BDNF in the amygdala of rodent models of adolescent alcohol exposure has recently been implicated in anxiety-like and alcohol-drinking behaviors^12^. Furthermore, mutant versions of BDNF with decreased TrkB activity are associated with increase in alcohol consumption.^14,15,18^. A particularly important downstream target of BDNF signaling is activity-regulated cytoskeleton-associated protein (ARC) which is a member of the immediate-early gene family and is important in synaptogenesis and synaptic plasticity as well as anxiety and alcohol intake phenotypes^19–21^. BDNF activates *ARC* expression through CREB and other transcription factor binding to the synaptic activity response element (SARE) site located in the *ARC* promoter approximately 7 kb upstream from the transcription start site^22,23^.

In addition to complex signaling, the *BDNF* gene also has a complex regulatory and genetic structure, with 9 different promoters encoding at least 20 different splice variants sharing one common exon that encodes the BDNF protein (known as exon IX)^13,24^ and expression of these variants are temporal- and region-specific^25^. Studies in animal models have revealed that different *BDNF* transcript variants are downregulated in the amygdala by adolescent alcohol exposure and associated with alcohol intake in adulthood^12^.

Long non-coding RNAs (lncRNAs) are broadly defined as RNAs that are greater than 200 bp and lack a functional open-reading frame. The majority of lncRNAs are found exclusively in the brain and are important in normal brain development, with temporal and regional specificity^26–28^. Most lncRNAs with described function are involved in regulating gene expression and chromatin structure through a number of different mechanisms including *cis*-tether, *trans*-regulation, allosteric modulators of epigenetic complexes (e.g. polycomb repressive complex II, PRC2) or transcription factors, or acting as a decoy either for transcription factors or repressors^29–31^. LncRNAs have recently been identified as being important in AUDs in adults^32^. Despite several advances in the understanding of lncRNA function, the function of the vast majority of lncRNAs are still unknown. A recent study characterized the role of the lncRNA *BDNF*-antisense (*BDNF-AS*) to be a negative regulator of BDNF exon IX expression and neurogenesis, and also described the overlap region between *BDNF* and *BDNF-AS*^33^. However, changes in *BDNF* and *BDNF-AS* expression and associated epigenetic regulatory mechanisms in the amygdala of human AUD subjects are currently unknown. We aimed to determine if there are epigenetic changes mediated by *BDNF-AS* that are operative in the regulation of BDNF expression in adolescence, and whether these changes persist into adulthood in the amygdala of human postmortem brains diagnosed with AUDs that began drinking before the age of 21. Here we report that the epitranscriptome and epigenetic mechanisms interact to regulate BDNF expression in the amygdala and are possibly involved in the pathophysiology of alcoholism that begins with drinking during adolescence.

## Materials and methods

### Samples

Human postmortem amygdala tissue was acquired from the New South Wales Brain Tissue Resource Center (Sydney, Australia). Criteria for inclusion of AUD samples were: age >18 years, no history of major psychiatric disorders, and no history of other substance abuse disorders. Demographic characteristics are provided in Supplementary Table 1. We have previously described this cohort^34^. Detailed methods are provided in Supplementary Information.

### Quantitative real-time PCR for mRNA levels

Quantitative real-time PCR (qPCR) was run using standard methods with either Taqman probes or specific primers (Supplementary Table 2). Changes in expression were determined using the ∆∆Ct method and normalized to mean Ct values of *ACTB* and *GAPDH*. Data are presented as average fold change of controls.

### Enzyme-linked immunosorbent assay for protein levels

BDNF protein was ascertained via the Quantikine ELISA total BDNF Assay (R&D Systems) following the manufacturer’s instructions. The optical density of each sample and standard was measured using the Spectra MR microplate reader (Dynex Technologies) and the amount of BDNF was calculated against the BDNF standard curve and expressed as pg/µg of total protein (Supplementary Methods).

### Chromatin immunoprecipitation assay

Chromatin immunoprecipitation (ChIP) was run following previously published protocols^35,36^. Purified DNA was analyzed by qPCR using primers (Supplementary Table 2). The data were analyzed using the ∆∆Ct method, normalizing to input, and the data are expressed as fold change relative to controls.

### RNA methylation immunoprecipitation (M6A RIP) assay

M6A RIP was performed using EpiMark® *N*6-methyladenosine Enrichment Kit (NEB) following the manufacturer’s instructions with some modifications. Purified RNA was sheared to <350 bp using a Covaris ME220 and then purified using Zymo RNA Clean and Concentrator 5 (Zymo) following the manufacturer’s instructions. Purified RNA was then measured for concentration using the Qubit BR RNA Assay (ThermoFisher Scientific) following the manufacturer’s instructions, and 1 µg was used for each pulldown with 2 µL of M6A antibody. Following 1 h incubation and washes, RNA was eluted and purified with MyOne Silane Beads (ThermoFisher Scientific) then prepared for reverse transcriptase in which 1 µL from pulldown or input was put in reverse transcriptase reaction. Resulting cDNA was then used for qPCR using primers designed to predict M6A sites located on *BDNF-AS* (Supplementary Table 2). M6A sites were predicted using SRAMP^37^. The data were analyzed using the ∆∆Ct method, normalizing to input, and the data are expressed as fold change relative to controls.

### Statistics

Statistical analysis was performed using SigmaStat (Systat Software, San Jose, CA, USA) and data were visualized using GraphPad Prism (La Jolla, CA, USA). Comparisons between groups were performed with Student’s *t*-tests. If comparisons failed normality tests then Mann–Whitney rank sum tests were performed. Pearson’s correlations were performed to determine linear correlation between two groups. Significance was set at *p* < 0.05.

## Results

### Expression of *BDNF* and *BDNF*-*AS* in the postmortem amygdala

Previous studies in rodents have demonstrated a decrease in BDNF expression after adolescent alcohol exposure in the amygdala^12^, but similar changes in the human amygdala have not yet been examined. In order to identify potential changes that are due to adolescent drinking we evaluated both a cohort that began drinking before the age of 21 (early onset) and a cohort who began drinking after the age of 21 (late onset). We then analyzed these two cohorts to see if there was differences in drinking or other demographics. We found that our early onset group drank 234 ± 56 g (mean ± SEM) of alcohol per day at time of death versus the late onset group which drank 157 ± 28 g of alcohol per day at time of death, although this observation was not statistically significant between early onset and late onset groups (Supplementary Table 1). We also found that there was no statistically significant differences in other metrics evaluated, including age, postmortem interval (PMI), total drinking years, and cigarette pack years (Supplementary Table 1).

**Fig. 1:**
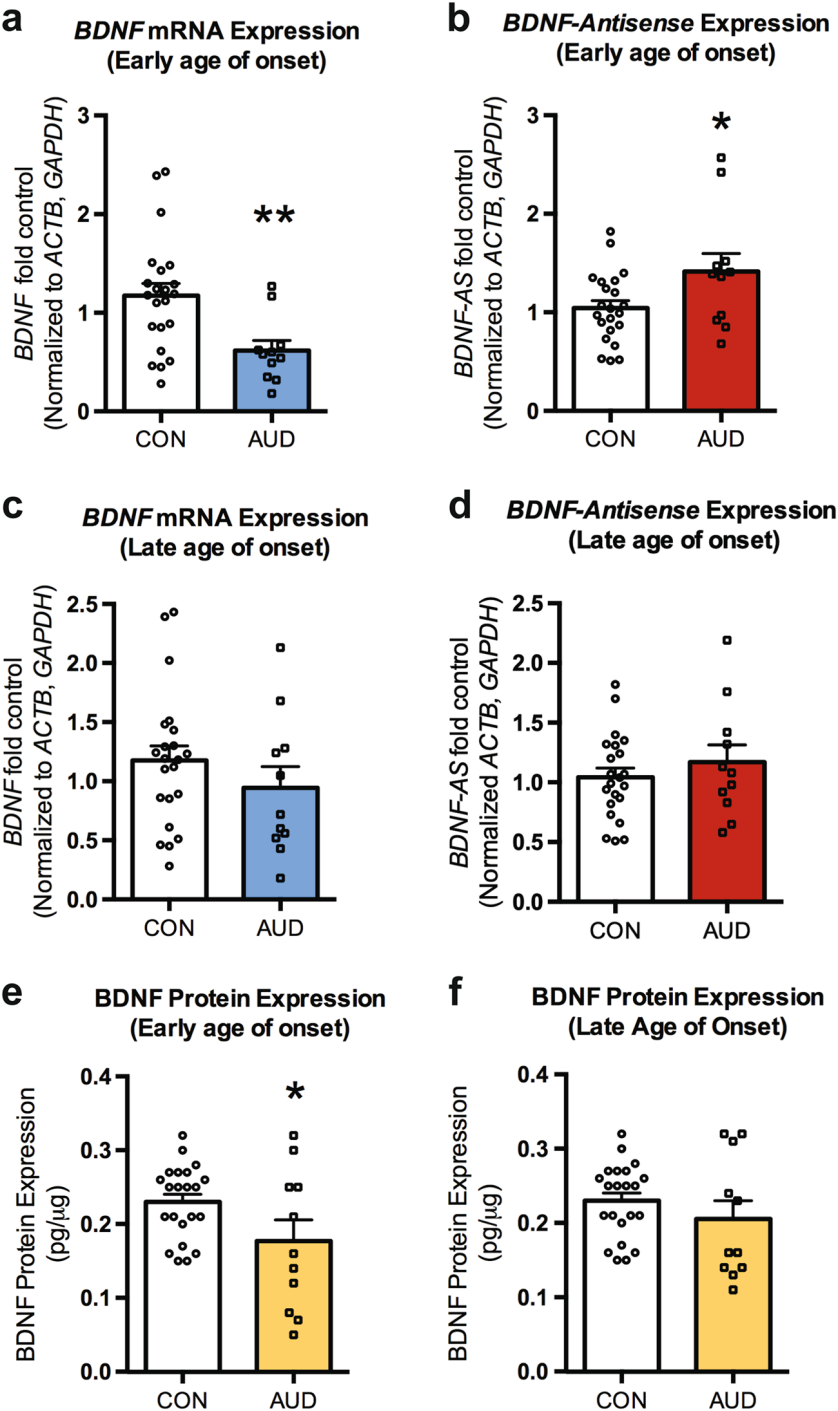
The early onset AUD individuals has increased BDNF-AS and decreased BDNF abundance in adult amygdala. **a** There is decreased *BDNF exon-IX* mRNA expression in the human amygdala of individuals with AUDs who began drinking before age of 21 (early onset). Significance was determined using Mann–Whitney test. **b** The lncRNA *BDNF-AS* is upregulated in the human amygdala of AUD patients who began drinking before the age of 21. **c** There is no change in *BDNF exon IX* mRNA expression in the human amygdala of individuals with AUDs who began drinking after age of 21 (late onset). **d** The lncRNA *BDNF-AS* is not changed in the human amygdala of AUD subjects who began drinking after the age of 21. **e** There is decreased BDNF protein levels in individuals with AUDs who began drinking in adolescence. Values are presented as mean ± SEM. **f** There is no change in BDNF protein levels in individuals with AUD who began drinking after adolescence. Values are presented as mean ± SEM. Significance was determined using Student’s *t*-test unless otherwise noted. *p < 0.05 and **p < 0.01. *n* = 22 (controls), 11 (early onset AUDs) and 11 (late onset AUDs)

We then evaluated *BDNF* mRNA expression at the common coding exon (*BDNF* exon IX) and found a significant decrease in *BDNF* mRNA expression in early onset AUD compared to controls (*p* = 0.008) (Fig. 1a). Regulation of *BDNF* expression is complex^25,26^; however, a previous study has demonstrated that increased *BDNF-AS* leads to decreased *BDNF* mRNA expression^33^. Therefore, we examined changes in the expression of lncRNA *BDNF-AS* in the postmortem amygdala of alcoholics who began drinking before the age of 21 to determine if this was a regulatory pathway potentially regulating decreased *BDNF* mRNA expression. *BDNF-AS* expression was increased in the early onset group (*p* = 0.033) (Fig. 1b). We also evaluated changes in both *BDNF* mRNA and *BDNF-AS* expression in the postmortem amygdala in the late onset group to determine if these changes were specific to early age onset group. There was no significant change in either *BDNF* mRNA (Fig. 1c) or *BDNF-AS* (Fig. 1d) expression in the late onset group.

We next analyzed correlations between *BDNF* and *BDNF-AS* expression versus changes in several drinking variables (total drinking years, alcohol daily use at time of death (in grams), and standard drinks per week) to determine if there was any relationship. We first analyzed the correlation between *BDNF* and *BDNF-AS* expression and found that *BDNF* and *BDNF-AS* mRNA levels are negatively correlated (*r* = −0.0443, *p* = 0.003). We then evaluated whether changes in *BDNF-AS* expression was a function of total drinking years, alcohol daily use at the time of death, and standard drinks per week. We found that *BDNF-AS* expression in total samples (*n* = 42) was positively correlated with alcohol daily use at time of death (*r* = 0.523, *p* = 0.0004) and standard drinks per week (*r* = 0.466 *p* = 0.002) but not total drinking years (Supplementary Fig. 1). Examination of AUD subjects who began drinking before the age of 21 revealed that *BDNF-AS* trended towards being significantly correlated with alcohol use per day at time of death (*r* = 0.567, *p* = 0.069), but this was not the case for controls (*r* = 0.212, *p* = 0.37) or in the late onset group (*r* *=* 0.258, *p* = 0.45) (Supplementary Fig. 1). Similarly, *BDNF* mRNA expression in total samples (*n* = 42) negatively correlated with alcohol per day use at time of death (*r* = −0.416, *p* = 0.0062) and standard drinks per week (*r* = −0.374 and *p* = 0.015) but not total drinking years (*r* = −0.253, *p* = 0.106) (Supplementary Fig. 1). When we determined correlations of *BDNF* mRNA expression separately versus standard drinks or alcohol daily use at time of death in only early onset drinker and late onset drinker groups we found no significant correlations. However, we did observe a trend towards significant correlation for *BDNF* and both alcohol use per day at time of death (*r* *=* −0.431, *p* = 0.058) and standard drinks per week (*r* *=* −0.43, *p* = 0.058) (Supplementary Fig. 1) for controls. We also found that there was no statistically significant correlations in other metrics evaluated: age, PMI, and cigarette pack years.

### BDNF protein levels in the postmortem amygdala

Concentration of total (free and TrkB bound) BDNF protein in postmortem human amygdala of AUDs and control subjects was determined via enzyme-linked immunosorbent assay. Average (mean ± SEM) BDNF protein (pg/μg of total protein) in controls (0.228 ± 0.011; *n* = 22) was not significantly different than total alcoholics (0.192 ± 0.019; *n* = 22). However, further analysis by separation of alcoholics by age of onset of drinking revealed a significant (*p* = 0.043) decrease in BDNF protein levels in subjects (*n* = 11) in the early onset group (0.177 ± 0.029) versus controls (0.228 ± 0.011; *n* = 22) (Fig. 1e). Interestingly, no significant (*p* = 0.31) differences were observed in the late onset group (0.207 ± 0.025; *n* = 11) versus controls (0.228 ± 0.011; *n* = 22) (Fig. 1f). BDNF protein levels in total samples (*n* = 42) were significantly negatively correlated with both standard drinks per week (*r* *=* −0.377, *p* = 0.014) and alcohol use per day at time of death (*r* = −0.376, *p* = 0.0142). When we determined correlation of BDNF protein expression separately in controls, early, and late of onset drinkers with standard drinks per week and alcohol use per day at time of death, we found no significant correlations similar to what was found with *BDNF* mRNA correlations (Supplementary Fig. 2).

### Epigenetic modifications at the BDNF gene in the postmortem amygdala

**Fig. 2:**
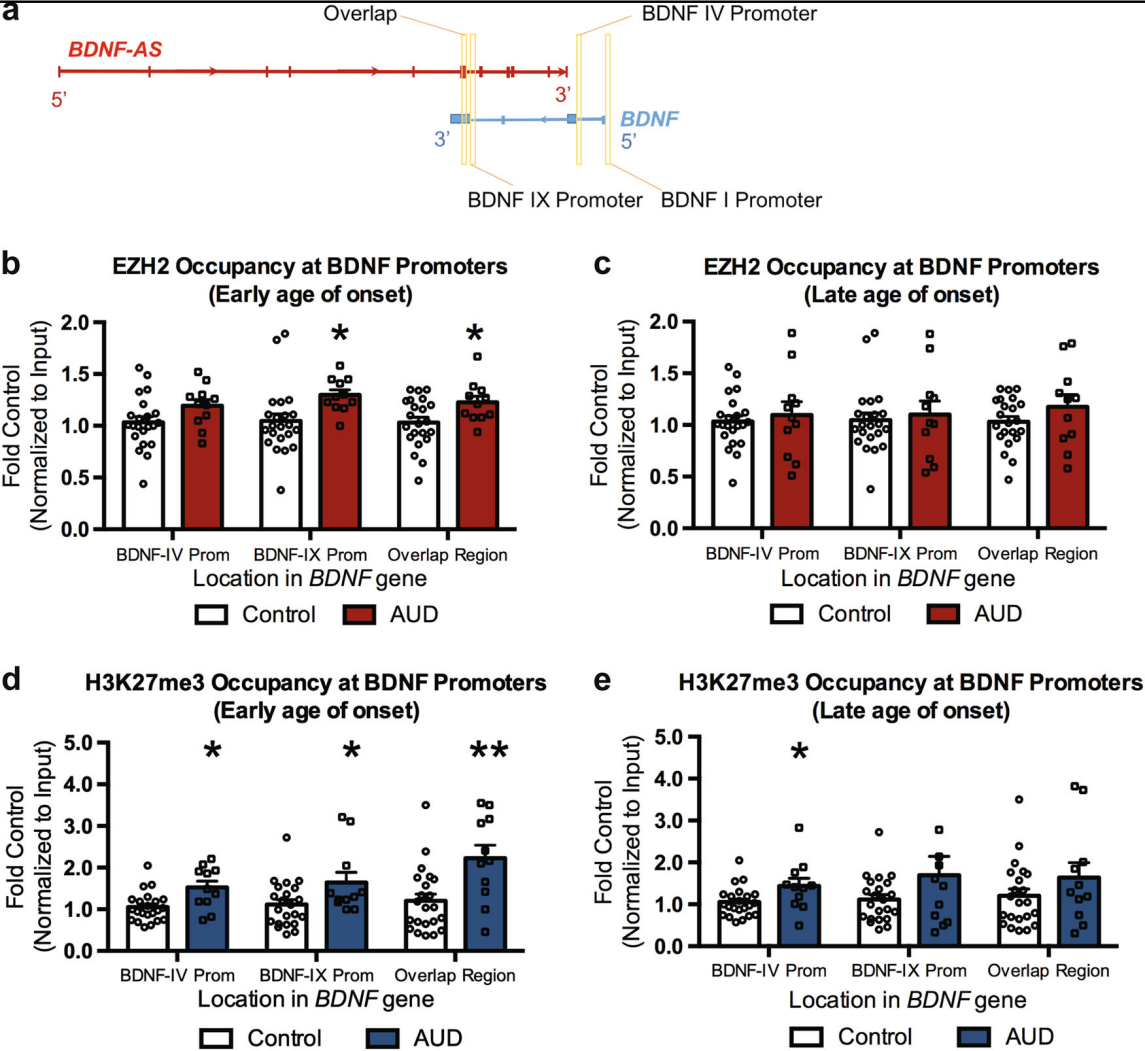
The early onset AUD individuals show repressive chromatin remodeling at several different BDNF gene loci. **a** Schematic demonstrating different *BDNF* sites evaluated for changes in occupancy of EZH2 and histone H3K27 trimethylation (H3K27me3). **b** EZH2 occupancy is elevated at the *BDNF-IX* promoter and the *BDNF* and *BDNF-AS* overlap site in the amygdala of individuals who began drinking before the age of 21. Significance was determined by Mann–Whitney test. **c** EZH2 is not elevated at any of the three *BDNF* sites measured in individuals who began drinking after the age of 21. Significance was determined by Mann–Whitney test for *BDNF-IV*, *BDNF-IX*, and *BDNF* and *BDNF-AS* overlap region in later age of onset. **d** Early onset in the postmortem amygdala of AUD subjects show increases in repressive H3K27me3 associated with several *BDNF* gene locations while **e** late onset show only increases in repressive H3K27me3 at the *BDNF-IV* promoter. Significance was determined by Mann–Whitney test for *BDNF-IV* for early age of onset and *BDNF-IX* for both early and late age of onset. Values are presented as mean ± SEM. Significance was determined using Student’s *t*-test unless otherwise noted. *p < 0.05 and **p < 0.01. AUD alcohol use disorder. *n* = 22 (controls), 11 (early onset AUDs) and 11 (late onset AUDs)

Previous reports in rodent models of adolescent alcohol exposure have demonstrated that changes in *BDNF* expression is due to changes in epigenetic modifications. Particularly, deficits in histone acetylation (H3K9/14ac) of *BDNF* exon IV promoter was associated with deficits in *BDNF* expression^12^. Further, *BDNF-AS* is known to contribute to epigenetic changes facilitated by the PRC2 complex at the *BDNF* exon IX promoter in HEK293 cells^33^. To evaluate if changes observed in *BDNF* expression were due to epigenetic changes, we utilized ChIP assay to determine if there were differences in enhancer of zeste homolog 2 (EZH2) occupancy and its catalytic product, repressive H3K27 trimethylation (H3K27me3), at several sites in the *BDNF* promoter region (Fig. 2a) and at a region where *BDNF exon IX* and *BDNF-AS* overlap^33^. We evaluated EZH2 occupancy at these loci. We observed increases in EZH2 at both the *BDNF-IX* promoter (*p* = 0.023) and overlap region (*p* = 0.027) in the early onset AUD group. The overlap region (Fig. 2a) is the region of overlap between transcribed *BDNF* and *BDNF-AS* transcripts (located in the common exon IX of *BDNF* and exon 5 of *BDNF-AS*)^33^. At the *BDNF-IV* promoter, though there appears to be a trend (*p* = 0.084) towards an increase in the early onset group (Fig. 2b). We observed no changes in EZH2 occupancy at any of the loci in the late onset group (Fig. 2c). There were significant increases in H3K27me3 associated with the *BDNF-IV* (*p* = 0.014) and *BDNF-IX* (*p* = 0.049) promoters and also at the overlap region (*p* = 0.003) (Fig. 2d) in the early onset group. In the late onset group, there was only an increase (*p* = 0.027) in H3K27me3 at the *BDNF-IV* promoter (Fig. 2e). We examined for correlations between repressive H3K27me3 at the BDNF-IX, promoter, and BDNF overlap region in total sample (*n* = 42) with drinking variables. We found no significant correlations with either alcohol use per day at the time of death nor standard drinks per week for either BDNF-IX and BDNF overlap region.

We next analyzed changes in *EZH2* and suppressor of zeste 12 (*SUZ12*, another member of the PRC2 complex) mRNA levels using qPCR to determine if changes in occupancy of EZH2 could be due to changes in overall levels. We found no change in either *EZH2* or *SUZ12* mRNA levels in early onset or late onset postmortem human amygdala (Supplementary Fig. 3). SUZ12 is a core component of the PRC2 complex and known binding partner of lncRNAs^30^. We therefore performed RIP assay to test for an interaction between *BDNF-AS* and SUZ12 protein and found that SUZ12 binds to *BDNF-AS* as determined by the RIP assay (Supplementary Fig. 4).

### Epigenetic modifications at the *ARC* SARE site and *ARC* mRNA levels in the postmortem amygdala

**Fig. 3:**
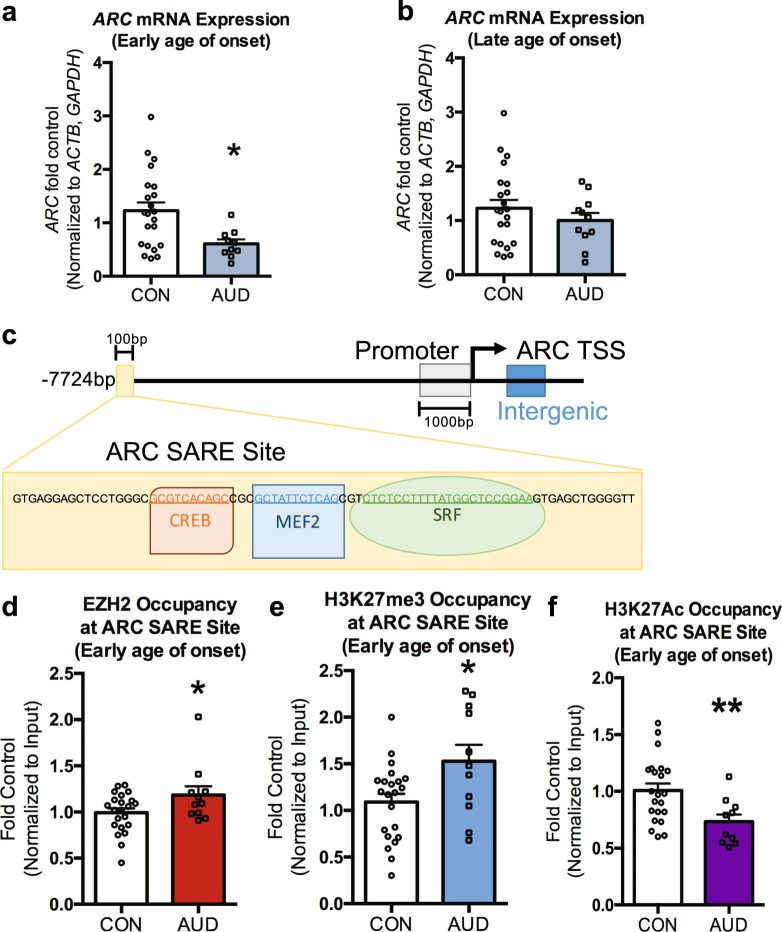
ARC, a downstream target of BDNF signaling, is decreased in the amygdala of early onset AUDs through interactions with the synaptic activity response element (SARE) in the ARC promoter. **a** *ARC* is decreased in the postmortem amygdala of individuals who began drinking during adolescence (early onset). Significance was determined using Mann–Whitney test. **b** *ARC* is not decreased in the postmortem amygdala individuals who began drinking in adulthood (late onset). **c** Representative schematic of the *ARC* SARE site which responds to BDNF activity and is located up ~7.7 kb upstream from the promoter. **d** Adolescent drinking increases EZH2 occupancy at the ARC SARE site in human postmortem amygdala. Values are presented as mean ± SEM. **e** Adolescent drinking increases repressive H3K27me3 associated with the ARC SARE site in human postmortem amygdala. **f** Adolescent drinking decreases H3K27ac associated with the ARC SARE site in human postmortem amygdala. Significance was determined using Student’s *t*-test unless otherwise noted. *p < 0.05 and **p < 0.01. *n* = 22 (controls), 10–11 (early onset AUDs) and 11 (late onset AUDs)

The early immediate gene *ARC* is a downstream target of BDNF, is involved in synaptic plasticity, and is a critical target of adolescent and adult alcohol exposure in rodent models^12,21^. Analysis of changes in *ARC* expression in early onset postmortem amygdala shows decreased *ARC* (*p* = 0.016) (Fig. 3a), but not in late onset postmortem amygdala (Fig. 3b). BDNF signaling is known to regulate *ARC* expression through the *ARC* SARE site located ~7.7 kb upstream of the transcription start site^22,23^ and is regulated by enzymes involved in epigenetic modulation^22,23^, and therefore we examined if there were epigenetic changes at this location (Fig. 3c). H3K27me3 occupancy was increased at the *ARC* SARE site (*p* = 0.018) in the early onset AUD group (Fig. 3d). We next evaluated if this change was mediated by increased PRC2 signaling and found increased EZH2 associated with the SARE site in this group as well (*p* = 0.049) (Fig. 3e). These changes are not present in the late onset AUD group (Supplementary Fig. 5**)**. We also found decreased H3K27ac (a histone post-translational modification associated with increased gene expression) associated with the SARE site in the early onset AUD group (*p* = 0.009) (Fig. 3f).

### RNA methylation of *BDNF-AS* in the postmortem amygdala

**Fig. 4:**
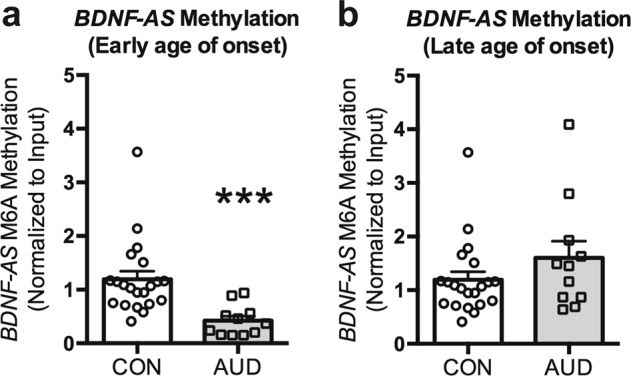
*BDNF-AS* is upregulated by a potential epitranscriptomic mechanism in early onset group. **a** Drinking during adolescence decreases RNA methylation at the predicted M6A site in *BDNF-AS*. **b** Drinking in adulthood does not change methylation at the M6A site in *BDNF-AS.* Values are presented as mean ± SEM. Significance was determined using Mann–Whitney rank sum test. ***p < 0.001. *n* = 21 (controls), 11 (early onset AUDs) and 11 (late onset AUDs)

We next wanted to determine the mechanism for *BDNF-AS* upregulation in early onset AUD subjects. Our initial experiments focused on H3K4me3 at the *BDNF-AS* promoter as this mark is known to be involved in active transcription and in the regulation of lncRNAs^38,39^. However we found no significant increase in H3K4me3 in the early onset group (Supplementary Fig. 6). Recent reports have suggested that post-translational modifications can modulate lncRNA expression^40^ so we utilized *N*6-methyladenosine (M6A) RIP assay to determine if there were changes in RNA methylation at predicted M6A sites in *BDNF-AS*. Our results indicate a decrease (*p* < 0.001) in *BDNF-AS* RNA methylation in the early onset AUD group (Fig. 4a), but not in the late onset AUD group (Fig. 4b). We did not observe any change in mRNA expression of several known RNA methyltransferases or the RNA demethylase *ALKBH5* (Supplementary Fig. 7a–h).

## Discussion

**Fig. 5:**
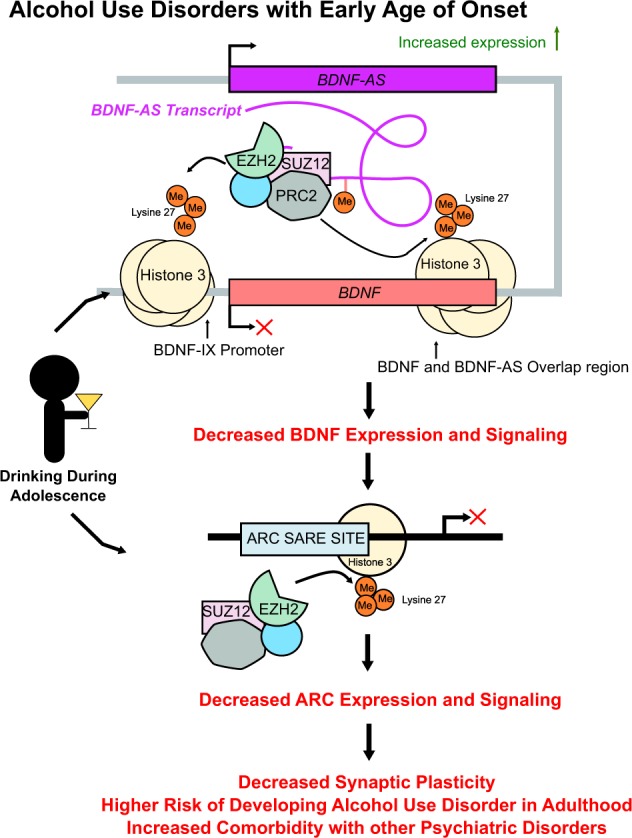
Proposed association of regulation of decreased *BDNF* expression by *BDNF-AS* through recruitment of the PRC2 complex in the human amygdala of individuals who began drinking in adolescence. Adolescent drinking appears to increase the expression of *BDNF-AS* most likely via inhibition of RNA methylation. This is associated with decreases in *BDNF* expression in the amygdala via recruitment of EZH2 and the associated increase in H3K27 trimethylation (H3K27me3) at the promoter and overlap region of *BDNF exon IX*. Interestingly, the reduction in *BDNF* expression is associated with a reduction in activity-regulated cytoskeleton-associated protein (*ARC)* expression, possibly via an increase in occupancy of EZH2 and H3K27me3 at an important regulatory site of the *ARC* gene known as synaptic activity response element (SARE). These *BDNF-AS-*regulated epigenetic mechanisms induced by adolescent drinking in humans appears to be important in the pathophysiology of alcohol use disorders (AUD) later in life

This study demonstrates that early age of onset of alcohol consumption increases *BDNF-AS* lncRNA expression induced via diminished RNA methylation, which triggers aberrant epigenetic mechanisms to produce deficits in BDNF signaling in the amygdala and possibly associated with pathophysiology of AUD (Fig. 5).

### Effects of adolescent drinking and changes in BDNF signaling in adulthood

BDNF signaling is an important modulator of CNS development through mediation of important processes such as neuronal survival, neurogenesis, neurite outgrowth, and neuronal differentiation^13,16^. Importantly, expression of both BDNF protein and specific *BDNF* mRNA isoforms are developmentally controlled, with different areas of the amygdala showing different expression patterns during adolescence and adulthood. BDNF expression in the central nucleus of amygdala (CeA) slowly rises to a plateau in adulthood, while the basolateral amygdala has decreased expression in adulthood relative to adolescence^41^ and disruption to normal BDNF expression and signaling in adolescence is likely to produce detrimental outcomes on normal brain development. Changes in BDNF signaling are likely to be involved in structural changes in the amygdala, which involve synaptic pruning and altered connectivity between the amygdala and other brain regions throughout the adolescent period^8^. Based on the previous finding that adolescent alcohol exposure in rodents decreases BDNF expression in the CeA^12^, our present findings confirm the hypothesis that there would be changes in BDNF expression in a cohort of postmortem amygdala from AUD subjects that began drinking during adolescence, demonstrating a conserved pathway that may induce increased drinking and other psychiatric problems in adulthood caused by adolescent alcohol exposure. Previous work has found that individuals with alcohol dependence have decreased circulating BDNF in blood^42^. Interestingly, transgenic mice harboring a homolog (Val68Met) of a common human polymorphism of BDNF (Val66Met) compulsively consume more alcohol than wild-type mice^18^. The Val68Met variant leads to decreased intracellular trafficking and activity-dependent BDNF release and function^18^. Decreasing BDNF or ARC expression in the CeA of rodent models of alcoholism in adulthood increases drinking and anxiety-like behaviors, which are measures of alcohol dependence^21,43^. These previous findings taken together with the present findings shown here suggest that disruptions to BDNF expression and ARC signaling are likely to increase the risk for alcohol dependence, and the risk is greater if these changes occur at a critical period of brain development^44^, such as adolescence, due to enduring effects on epigenetic programming. However, the possibility cannot be ruled out that other factors besides drinking during adolescence are involved with changes in BDNF expression that we observed in human postmortem amygdala. These factors could include a longer time period for disruption of normal neural processes by alcohol and a higher lifetime alcohol consumption at the time of death.

### Potential role of lncRNAs in epigenetic regulation by adolescent drinking in AUD subjects

LncRNAs have recently emerged as a potential molecular target in AUDs by the use of RNA sequencing^32,45^. However, evaluation of lncRNAs in a population of individuals with AUDs that began drinking before the age of 21 has not been explored. Previous studies have characterized the lncRNA, *BDNF-AS*, to be a negative regulator of BDNF expression^33^. Rodent studies have demonstrated that infusion of *BDNF*-antisense oligodeoxynucleotides into the CeA increases anxiety and alcohol consumption^43^. Therefore, we postulated that the naturally occurring *BDNF-AS* may be involved in changes observed in BDNF expression in our early onset cohort. We found increased *BDNF-AS* only in individuals who began drinking before the age of 21. Additionally, similar to previous findings demonstrated in vitro^33^, increased *BDNF-AS* negatively correlated with *BDNF exon IX* expression. Intriguingly, we also found that *BDNF-AS* expression positively correlated with the amount of alcohol consumed using two different metrics of alcohol consumption (grams consumed per day at time of death and standard drinks per week). One possibility is that earlier age of alcohol use may interact with later alcohol consumption to induce *BDNF-AS* expression. Our results suggest that *BDNF-AS* may also be important in epigenetic programming during development, and expression of this transcript is dysregulated after drinking in adolescence and associated with AUDs later in life.

Our data indicate that *BDNF-AS* may be regulated epitranscriptomically by decreased RNA methylation at a M6A site in the amygdala of early onset AUD but not in late onset AUD. Previous reports have found that decreased M6A methylation prevents RNA turnover and decreases targeted degradation by YTH domain family 2, which is recruited to the RNA by M6A methylation^46^. Other roles for M6A methylation appear to either promote or inhibit recruitment of proteins that bind RNA^47^, a potential mechanism for the binding of SUZ12 to *BDNF-AS* and could be explored in future experiments. Nonetheless, the observation that adolescent alcohol exposure decreases *BDNF-AS* methylation in the amygdala reveals new mechanisms of RNA regulation that add another layer of complexity of epigenetic regulation of BDNF expression in AUD.

### Role of PRC2 complex regulating epigenetic changes in early onset AUD

LncRNAs have been demonstrated to interact with SUZ12, a member of the PRC2 complex, via its zinc-finger domain^30^, and *BDNF-AS* knockdown disrupts EZH2 recruitment to *BDNF* promoters in cell culture models^33^. We tested this hypothesis by examining EZH2 occupancy and its catalytic product, H3K27me3, at regions of the *BDNF* promoter, including the region of *BDNF exon IX* that overlaps with *BDNF-AS*. Our results indicate that there is increased EZH2 recruited to both the *BDNF* overlap region and the promoter of *BDNF exon IX* with corresponding increases in H3K27me3 at these locations. Coupled with our data showing that SUZ12 binds directly to *BDNF-AS*, this suggests that *BDNF-AS* regulates *BDNF-IX* expression through direct binding and recruitment of the PRC2 complex and subsequent repression of transcription by deposition of H3K27me3.

In this study, we also found increases of EZH2 and H3K27me3 at the *ARC* SARE site. This may be due to chromatin remodeling secondary to decreased BDNF signaling and recruitment of class I histone deacetylases (HDACs)^22^ catalyzing removal of histone acetyl groups, which we have also observed. The recruitment of EZH2 to this site warrants further experimentation to determine if it occurs through decreased BDNF signaling, subsequently decreased occupancy of CREB-binding protein, and increased occupancy of class I HDACs at the SARE site of *ARC* gene. EZH2 is also heavily implicated in brain development, where it has a role in regulating self-renewal and differentiation in the cerebral cortex^48^. Since there is significant pruning and differentiation of neurons during adolescence^44^, EZH2 dysregulation and an increase in repressive H3K27me3 marks at the *BDNF* and *ARC* regulatory regions by alcohol drinking during this critical period could contribute to an increased risk of AUDs and higher overall alcohol consumption later in life (Fig. 5).

## Conclusions

Our results appear to suggest that *BDNF-AS* is upregulated most likely via decreased RNA methylation in the amygdala of adult AUD population who began drinking during adolescence, but not in AUD subjects who began drinking in adulthood. However we are unable to determine if these effects are causative without further experimentation, and indeed changes that we observed could be due to other factors and interactions. Future experiments will have to consider these possibilities in larger cohorts of human postmortem brain and through the use of reverse translational approach in animal models. We demonstrate that increased *BDNF-AS* is associated with decrease in *BDNF* expression through an epigenetic mechanism involving the recruitment of PRC2 and the repression of gene promoters. Downstream signaling targets of BDNF such as *ARC* are also decreased through recruitment of EZH2 and the deposition of repressive H3K27me3 marks. Our results provide translational evidence for a mechanism for decreased BDNF in the amygdala previously shown in rodent models of adolescent alcohol exposure: namely, similar decreases in *BDNF* and *ARC* that are associated with higher alcohol intake^12^. These results suggest the possible role for developmentally sensitive lncRNAs in early onset AUD, and that EZH2 inhibitors may prove useful in the treatment of adult psychopathology after adolescent alcohol drinking.

## Supplementary information

Supplemental Materials
