# Supplementary material for: The lncRNA BDNF-AS is an epigenetic regulator in the human amygdala in early onset alcohol use disorders
Source: Transl Psychiatry. 2019 Feb 6;9:34. doi: 10.1038/s41398-019-0367-z (PMC6365546; doi:10.1038/s41398-019-0367-z)
Supplement: Supplementary file 1 — Supplemental Materials [file 41398_2019_367_MOESM1_ESM.docx]

**Supplementary Methods**

**RNA extraction**

RNA was extracted from the amygdala using miRNeasy Mini Kit (Qiagen, Hilden, Germany) following manufacturer’s instructions. RNA concentration was determined using a Nanodrop 2000 (ThermoFisher Scientific, Waltham, MA, USA) and RNA integrity was determined using the Agilent 4200 TapeStation (Agilent, Santa Clara, CA, USA).

**Quantitative real-time PCR (qPCR)**

Total RNA was reverse transcribed into cDNA using MultiScribe Reverse Transcriptase (ThermoFisher Scientific, Waltham, MA, USA) following manufacturer’s instructions. qPCR reactions were run on a CFX-Connect qPCR system using TaqMan™ Fast Advanced Master Mix and the following TaqMan probes: *BDNF-AS*, Hs01010228_m1; *BDNF,* Hs02718934_s1;*GAPDH*, Hs03929097_g1; *ACTB*, Hs01060665_g1 (ThermoFisher Scientific, Waltham, MA, USA). *ARC, EZH2*, *SUZ12, ALKBH5, METTL3, METTL14,* and *METTL16* expression were analyzed using specific primers (**Supplementary** **Table 2**) and SsoAdvanced^TM^ universal SYBR green super mix (BioRad, Des Plaines, IL, USA). Changes in expression were determined using the ∆∆Ct method^1^ and normalized to mean of Ct values of *ACTB* and *GAPDH*, housekeeping genes. Data are presented as average fold change relative to controls.

**ELISA**

Frozen human amygdala tissue was homogenized in RIPA buffer (Pierce, Rockford, IL) plus protease inhibitors (Santa Cruz, Dallas, TX). Homogenates were incubated on ice for 30min and then centrifuged at 17,000×g for 20min at 4°C. Supernatant was collected and protein content was measured employing the BCA Protein Assay Kit (Pierce, Rockford, IL). BDNF protein was then ascertained via the Quantikine ELISA Total BDNF Assay (R&D systems, Minneapolis, MN). Optimal protein concentration was determined through an initial a protein curve and therefore 150µg of protein per sample was used. All samples were run in duplicate and replicated across a minimum of two ELISA assays. The optical density of each sample and standard was measured using the Spectra MR microplate reader (Dynex Technologies, Chantilly, VA) and the amount of BDNF was calculated against the BDNF standard curve and expressed as pg/µg of total protein.

**Chromatin Immunoprecipitation (ChIP)**

Chromatin immunoprecipitations were performed as previously described^2^. Frozen amygdala tissue was fixed in 1% methanol-free formaldehyde at room temperature for 10 min. Crosslinking was quenched with 1M glycine in 750 mM Tris HCl before homogenization in lysis buffer (1% (v/v) SDS, 10 mM EDTA, 50 mM Tris-HCl pH 8.0). Homogenate was then sonicated using the Covaris ME220 (Covaris, Woburn, MA, USA) to achieve sheared DNA fragments of 200-500 base pairs which were then clarified using centrifugation (17,000×g for 10 min, 4°C) to obtain a chromatin fraction. Chromatin concentration was then determined using the Qubit™ dsDNA HS Assay Kit (Thermo Fisher Scientific, Waltham, MA, USA). 1μg chromatin (based on DNA concentration) was then diluted in ChIP washing buffer (0.01% SDS, 1.1% Triton X-100, 1.2 mM EDTA, 16.7 mM Tris-HCl pH 8.0, 167 mM NaCl) and an aliquot was taken for input normalization. The remaining sonicated chromatin was incubated overnight at 4ºC with either H3K27me3 (Active Motif, Cat# 39155; 2µg) H3K27Ac (Active Motif, Cat# 39133, 2µg), H3K4me3 (Cell Signaling, Cat# 9727, 2µg), and EZH2 (Active Motif, Cat# 39875; 2µg) antibody. Magnetic Protein A Dynabeads (ThermoFisher Scientific, Waltham, MA, USA) were then added to chromatin samples and rotated for 1hr 30min at 4°C. Chromatin was then washed five times using washing buffer prior to DNA purification using Chelex-100 resin (10% w/v), Bio-Rad) by boiling at 95°C for 10 min. The input aliquot was precipitated using 100% ethanol, washed once with 75% ethanol and then purified by using (10% w/v) Chelex-100 resin at 95°C for 10 min. Proteinase K (20 mg/mL, final concentration 0.4 µg/µL, ThermoFisher Scientific) was then added both pulldown and input samples and incubated at 55ºC for 1hr. Samples were then incubated at 95ºC for 10min to inactivate Proteinase K and centrifuged at 7,000×g for 2min. Purified DNA was analyzed by quantitative PCR using primers (**Supplementary** **Table 2**). The data was analyzed using the ∆∆Ct method^1^, normalizing to input, and the data are expressed as fold change in protein occupancy.

**RNA Immunoprecipitation (RIP)**

RIP was performed as previously described^3^ with some modifications. Amygdala tissue was dissected and homogenized in nuclear extraction buffer (0.32M sucrose, 10mM Tris-HCl pH 7.5, 5 mM MgCl2, and 1% Triton X-100) then left on ice for 30 minutes. Tissue was pelleted at 2,500×g at 4ºC for 15 minutes, then pellet was resuspended in RIP buffer (150 mM KCl, 25 mM Tris pH 7.4, 5 mM EDTA, 0.5 mM DTT, 0.5% NP40 with protease (Halt™ Thermoscientific, Walathm, MA, USA) and RNase Inhibitors (100 units/mL). RNA was fragmented using the Covaris ME220 (Covaris, Woburn, MA, USA) then pelleted by centrifugation at 13,000×g at 4ºC for 10 minutes. RNA concentration in clarified supernatant was measured using Qubit™ RNA HS Assay Kit (Thermo Fisher Scientific, Waltham, MA, USA). SUZ12 antibody (#39357, Active Motif, 2µg) was added to 1µg of RNA after 100ng was removed for input, then incubated overnight at 4°C. Magnetic Protein A Dynabeads (ThermoFisher Scientific, Waltham, MA, USA) were then added to chromatin samples and rotated for 1hr at 4°C. Beads were washed 3× with RIP buffer, then once with PBS and isolated by the addition of Qiazol reagent (Qiagen, Germantown, MD, USA). Both pulldown and input were purified using a miRNAeasy Minikit (Qiagen, Germantown, MD, USA) then reverse transcribed using MultiScribe Reverse Transcriptase (ThermoFisher Scientific, Waltham, MA, USA) following manufacturer’s instructions and analyzed using qPCR with primers specific for *BDNF-AS* (**Supplementary** **Table 2**). PCR reactions were then analyzed on an Agilent 4200 TapeStation (Agilent, Santa Clara, CA, USA) for specificity.

**Supplementary Figures 1-7**

**Supplementary Figure 1. Correlations between *BDNF* or *BDNF-AS* and standard drinks per week or alcohol daily use at time of death. a)** *BDNF-AS* is positively correlated with number of standard drinks per week. **b)** *BDNF-AS* is positively correlated with alcohol use at time of death in (grams/day). **c)** *BDNF* is negatively correlated with number of standard drinks per week. **d)** *BDNF* is negatively correlated with alcohol use at time of death in (grams/day). Statistical significance was determined using Pearson’s correlations. n = 20 (Control), n = 11 (Early onset AUDs), n = 11 (Late onset AUDs). 2 controls did not have drinking data and were excluded.

**Supplementary Figure 2. Correlations between BDNF protein and standard drinks per week or alcohol daily use at time of death. a)** BDNF protein is negatively correlated with number of standard drinks per week. **b)** BDNF protein is negatively correlated with alcohol use at time of death in (grams/day). Statistical significance was determined using Pearson’s correlations. n = 20 (Control), n = 11 (Early onset AUDs), n = 11 (Late onset AUDs). 2 controls did not have drinking data and were excluded.

**Supplementary Figure 3. There is no change in expression of PRC2 complex components *EZH2* and *SUZ12* in postmortem human amgydala in either early onset or late onset AUDs. a)** *EZH2* expression as measured by qPCR is not significantly different between the control subjects and early onset AUDs. **b)** *EZH2* expression as measured by qPCR is not significantly different between the control subjects and late onset AUDs. **c)** *SUZ12* expression as measured by qPCR is not significantly different between the control subjects and early onset AUDs. **d)** *SUZ12* expression as measured by qPCR is not significantly different between the control subjects and late onset AUDs. Values are presented as mean ± SEM. Statistical significance was determined by Student’s t-test. n = 22 (Control), n = 11 (Early onset AUDs), n = 11 (Late onset AUDs except *SUZ12* where n=10).

 **Supplementary Figure 4. PRC2 complex binds directly to BDNF-AS in the amygdala of control and AUD subjects.** **a)** RNA immunoprecipitation (RIP) reveals direct interaction of *BDNF-AS* and SUZ12, a component of the PRC2.

**Supplementary Figure 5. EZH2 nor H3K27me3 is changed in late onset group (AUD) at the *ARC* SARE site. a)** Chromatin immunoprecipitation reveals that there is no increase in EZH2 at the *ARC* SARE site in the amygdala of individuals who began drinking after the age of 21. **b)** Chromatin immunoprecipitation reveals that there is no significant change in H3K27me3 at the *ARC* SARE site in individuals who began drinking after the age of 21. Values are presented as mean ± SEM. Statistical significance was determined by Student’s t-test. n = 22 (Control), n = 11 (Late onset AUDs).

**Supplementary Figure 6. H3K4me3 is not increased at the *BDNF-AS* promoter.** ChIP analysis shows no change in H3K4me3 associated with the *BDNF-AS* promoter in early age of onset AUDs in postmortem amygdala. Values are presented as mean ± SEM. Statistical significance was determined by Student’s t-test. n = 11 (Control), n = 11 (Early onset AUDs),

**Supplementary Figure 7.** **a-h)** qPCR analysis reveals that there is no significant change in *METTL3, METTL14, METTL16,* or *ALKBH5* in early onset or late onset AUDs in human postmortem amygdala. Values are presented as mean ± SEM. Statistical significance was determined by Student’s t-test or Man-Whitney rank sum test. n = 22 (Control), n = 11 (Early onset AUDs), n = 11 (Late onset AUDs).

**Supplementary Table 1.** Detailed demographic characteristic of subjects

| **Subject Characteristic** | **Controls** | **AUD onset before 21** | **AUD onset after 21** |
| --- | --- | --- | --- |
| N | 22 | 11 | 11 |
| Age | 58 ± 2 | 55 ± 2 | 59 ± 2 |
| Sex M/F | 18 / 4 | 11 / 0 | 7 / 4 |
| PMI (hr) | 33 ± 3 | 39 ± 4 | 31 ± 4 |
| pH | 6.66 ± 0.05 | 6.63 ± 0.06 | 6.59 ± 0.08 |
| Amygdala RIN | 5.08 ± 0.19 | 5.48 ± 0.39 | 4.55 ± 0.32 |
| BMI | 30 ± 1.3* | 26 ± 2.0 | 26 ± 1.6 |
| Total Drinking Years | 30 ± 4** | 37 ± 2 | 31 ± 3 |
| EtOH daily use (gm) | 16 ± 4** | **234 ± 56** | **157 ± 28** |
| Standard drinks per week | 10 ± 3** | **131 ± 38** | **73 ± 14** |
| Pack Years (Cigarettes) | 25 ± 7*** | 33 ± 4**** | 43 ± 10**** |
| Key: PMI = postmortem interval, RIN = RNA integrity number, Pack years = number of packs a person has smoked per day for the number of years a person has smoked. | | | |
|  |  |  |  |
| Values in bold are statistically different than controls [Standard drinks/week, Kruskal-Wallis one way analysis followed by Dunn’s test, control vs AUD onset before 21, p<0.05; control vs AUD onset after 21, p<0.05; EtOH daily use, Kruskal-Wallis one way analysis followed by Dunn’s test, control vs AUD onset before 21, p<0.05; control vs AUD onset after 21, p<0.05].  *n = 21. ** n = 20. *** n = 13. **** n = 10. Rest of subject data not available in cohort. Values are presented as mean ± SEM. | | | |
|  |  |  |  |

| **Supplementary Table 2.** Primers | |
| --- | --- |
| **Chromatin Immunoprecipitation** |  |
| BDNF-IV Promoter_Forward | CTGGTAATTCGTGCACTAGAGT |
| BDNF-IV Promoter_Reverse | CACGAGAGGGCTCCACGGT |
| BDNF-IX Promoter_Forward | CACTTGCAGTTGTTGCTTA |
| BDNF-IX Promoter_Reverse | GGCTTCAAGTTCTCCTTCTTCCCA |
| BDNF-IX Overlap_Forward | GAGCATCACCCTGGACGTG |
| BDNF-IX Overlap_Reverse | GCTTGACATCATTGGCTGACA |
| ARC-SARE_Forward | ACAGCCGCGCTATTCTCAG |
| ARC-SARE_Reverse | CGTCCAGGACTGTACGCTG |
| BDNF-AS Promoter_Foward | GGTGTACGGGTCTCCGAATG |
| BDNF-AS Promoter_Reverse | TCACACGGACTGTTCCAAGG |
| **qPCR** |  |
| ARC_Forward | GGTTCATCGTTCTGCCTTGT |
| ARC_Reverse | CCAGCCTTGAGGATTGGTTATG |
| EZH2_Forward | ACATCCTTTTCATGCAACACC |
| EZH2_Reverse | TTGGTGGGGTCTTTATCCGC |
| SUZ12_Forward | GAAGCCGAAAATGGAGCACG |
| SUZ12_Reverse | CTGTGTTGGCTTCTCAAAGGC |
| METTL3_Foward | TCTGGGGGTATGAACGGGTA |
| METTL3_Reverse | CTGGTTGAAGCCTTGGGGAT |
| METTL16_Forward | TCAATTGGAAGCCAAGGGAGT |
| METTL16_Reverse | ACCCCTTGTATGCGAAGCTC |
| METTL14_Forward | GTAGCACAGACGGGGACTTC |
| METTL14_Reverse | GAGCCAGCCTGGTCGAATTG |
| ALKBH5_Foward | CCCCATCCACATCTTCGAGC |
| ALKBH5_Reverse | ATCAGCAGCATATCCACTGAGC |
| GAPDH_Forward | CGAGATCCCTCCAAAATCAA |
| GAPDH_Reverse | TTCACACCCATGACGAACAT |
| ACTB_Forward | CTCCCTGGAGAAGAGCTAC |
| ACTB_Reverse | GATCCACACGGAGTACTTG |
| **RNA Immunoprecipitation** |  |
| BDNF-AS_Met_Forward | GACCACAGAGACAGCAGAACA |
| BDNF-AS_Met_Reverse | GGCTCTTGTTGGCACAGTCT |

**References**

1. Livak, K.J.& Schmittgen, T.D. Analysis of relative gene expression data using real-time quantitative PCR and the 2(-delta delta C(T)) method. *Methods* **25**(4), 402-408 (2001).
2. Zhang, H., Kyzar, E. J., Bohnsack, J. P., Kokare, D. M., Teppen, T. & Pandey, S. C. Adolescent alcohol exposure epigenetically regulates CREB signaling in the adult amygdala. *Scientific Reports* **8,** 10376 (2018).
3. Khalil, A. M., Guttman, M., Huarte, M., Garber, M., Raj, A., Rivea Morales, D., *et al.* Many human large intergenic noncoding RNAs associate with chromatin-modifying complexes and affect gene expression. *Proc Natl Acad Sci U A* **106,** 11667–11672 (2009).
